# Medicines for Malaria Venture Pandemic Box In Vitro Screening Identifies Compounds Highly Active against the Tachyzoite Stage of *Toxoplasma gondii*

**DOI:** 10.3390/tropicalmed8120510

**Published:** 2023-11-29

**Authors:** Mike dos Santos, Andréia Luiza Oliveira Costa, Guilherme Henrique de Souza Vaz, Gabriela Carolina Alves de Souza, Ricardo Wagner de Almeida Vitor, Érica S. Martins-Duarte

**Affiliations:** 1 Laboratório de Quimioterapia de Protozoários Egler Chiari, Departamento de Parasitologia, Instituto de Ciências Biológicas, Universidade Federal de Minas Gerais, Belo Horizonte 31270-901, Brazilgh.vaz7@gmail.com (G.H.d.S.V.);; 2 Laboratório de Toxoplasmose, Departamento de Parasitologia, Instituto de Ciências Biológicas, Universidade Federal de Minas Gerais, Belo Horizonte 31270-901, Brazil; ricardovitor@icb.ufmg.br

**Keywords:** toxoplasmosis, drug repositioning, new therapies, treatment, ultrastructure

## Abstract

Toxoplasmosis is a disease that causes high mortality in immunocompromised individuals, such as AIDS patients, and sequelae in congenitally infected newborns. Despite its great medical importance, there are few treatments available and these are associated with adverse events and resistance. In this work, after screening the drugs present in the Medicines for Malaria Venture Pandemic Box, we found new hits with anti-*Toxoplasma gondii* activity. Through our analysis, we selected twenty-three drugs or drug-like compounds that inhibited the proliferation of *T. gondii* tachyzoites in vitro by more than 50% at a concentration of 1 µM after seven days of treatment. Nineteen of these compounds have never been reported active before against *T. gondii*. Inhibitory curves showed that most of these drugs were able to inhibit parasite replication with IC_50_ values on the nanomolar scale. To better understand the unprecedented effect of seven compounds against *T. gondii* tachyzoites, an ultrastructural analysis was carried out using transmission electron microscopy. Treatment with 0.25 µM verdinexor, 3 nM MMV1580844, and 0.25 µM MMV019724 induced extensive vacuolization, complete ultrastructural disorganization, and lytic effects in the parasite, respectively, and all of them showed alterations in the division process. Treatment with 1 µM Eberconazole, 0.5 µM MMV1593541, 1 µM MMV642550, 1 µM RWJ-67657, and 1 µM URMC-099-C also caused extensive vacuolization in the parasite. The activity of these drugs against intracellular tachyzoites supports the idea that the drugs selected in the Pandemic Box could be potential future drugs for the treatment of acute toxoplasmosis.

## 1. Introduction

Toxoplasmosis, caused by the protozoan *Toxoplasma gondii*, is one of the most important zoonoses in the world, showing high prevalence and potential for morbidity and mortality. It is estimated that one third of the human population is infected by *T. gondii* [1]. However, in countries such as Brazil, the percentage of the population that has had contact with the parasite represents 42 to 92% [2]. In most individuals, the infection is asymptomatic, but in immunocompromised patients, it can progress to chorioretinitis, encephalitis, and other involvements in the Central Nervous System (CNS). Toxoplasmosis is one of the most frequent opportunistic diseases in the acquired immunodeficiency syndrome (AIDS) caused by the Human Immunodeficiency Virus (HIV), and about one third of all CNS affections in this population are due to toxoplasmosis [3,4]. Infections in pregnant women could lead to serious consequences for the fetus, such as abortion, microcephaly, anencephaly, blindness, chorioretinitis, and other neurological and ophthalmological sequelae [5]. South America has a higher rate of sequelae in newborns caused by incorporated toxoplasmosis than other regions of the world [6], only in Brazil; the incidence of congenital transmission of *T. gondii* can reach 1:770 live births in Brazil [7].

Despite the medical importance of toxoplasmosis, there are few drugs available for the treatment of this protozoosis. The administration of the triple association of pyrimethamine (PYR), sulfadiazine (SDZ), and folinic acid is the first choice for most clinical conditions of toxoplasmosis. The few replacements available include clindamycin (second line in replacement of SDZ), spiramycin (pregnant individuals at first-semester gestation), sulfamethoxazole, trimethoprim (in replacement of SDZ-PYR when not available), atovaquone, and azithromycin. Indeed, the therapeutic schemes present important weaknesses such as side-effects frequently reported by patients who abandon the treatment [8,9,10]; first-line therapy is potentially teratogenic (PYR); there is no treatment against the chronic phase of the disease; and mainly, therapeutic failures or relapses are frequently reported [10], which suggests the existence of resistant strains of *T. gondii* [11]. Thus, a new promising drug for toxoplasmosis should be well tolerated, safe for use in pregnant women, active against the acute and chronic phases of the disease, show a simplified administration and posology, and, due to the social factors that permeate this pathology, should have low production and acquisition costs.

The development of new drugs and treatments that are economically viable, safe, and effective for the treatment of parasitic diseases is one of the main current challenges. Although there are discussions in the academic sphere, we can frame toxoplasmosis in neglected parasitic infections, since there is no interest in the production of new therapeutic compounds by the pharmaceutical industry, due to the low expected financial return [12]. In this context, the repositioning of existing compounds and drugs is a promising strategy for the availability of new treatments [13,14].

Repositioning is defined as a strategy that seeks new therapeutic applications for an existing drug in the market or at the development stage. Thus, it has the advantage of ensuring a new effective treatment with economic viability, since these are molecules that are available in the pharmaceutical market [13,14,15]. Based on this, and with the aim to enhance the discovery of new treatments for infectious diseases, the Medicines for Malaria Venture (MMV) organization develops and makes available drug libraries containing promising new compounds and drugs with repositioning potential for the treatment of neglected infectious diseases with the potential to cause epidemics due to antimicrobial resistance. The screening of broad drug bases has proven to be an effective and rapid tool for the development of new treatments [16]. MMV has launched approximately five boxes. Boyom et al. (2014), Subramanian et al. (2018), and Varberg et al. (2018) demonstrated the anti-*T. gondii* activity of the compounds available in Malaria Box [17,18,19]. Spalenka et al. (2018) and Radke et al. (2018) identified, respectively, 25 and 82 Pathogen Box compounds that inhibit *T. gondii* replication [20,21]. Cajazeiro et al. (2022) and Santos et al. (2023) demonstrated the activity of COVID Box compounds against *T. gondii* [22,23]. Pandemic Box compounds were not previously available in Malaria or Pathogen Boxes. The last box made available by MMV was the Global Health Priority Box in 2021, with 260 compounds that could be analyzed in the coming years. In addition, through the Malaria Box and Pathogen Box, researchers from all over the world have found promising compounds for the treatment of diseases caused by *Cryptococcus neoformans* and *Candida albicans* [24,25]; *Schistosoma mansoni* [26]; *Giardia* and *Cryptosporidium* [27]; *Plasmodium falciparum* [28]; and even *T. gondii* [20].

In line with this, in 2019, MMV launched the Pandemic Box. This box was assembled with a set of 400 drug and drug-like molecules with previous known effects against bacteria (201 antibiotics), viruses (153 antivirals), and fungi (46 antifungals). In this context, the present study sought new treatments against the acute stage of *T. gondii* by evaluating the activity of 400 compounds contained in the MMV Pandemic Box present in this drug library.

## 2. Materials and Methods

### 2.1. Tested Compounds of Pandemic Box

The Pandemic Box was provided free of charge by the organization MMV (https://www.mmv.org/mmv-open/pandemic-response-box). The 400 compounds were supplied in five plates (A to E) containing 80 compounds each, at a volume of 10 µL and in a concentration of 10 mM in DMSO. After arrival, compounds were dissolved to a final concentration of 2 mM in DMSO (Merck).

### 2.2. Plaque Assay

For the preliminary evaluation of the 400 compounds contained in the Pandemic Box, 6-well plates were seeded with neonatal Normal Human Dermal Fibroblast cells (NHDF; Lonza, kindly donated by Dr. Sheila Nardelli, Fiocruz Paraná, Brazil) in RPMI 1640 medium supplemented with 2% Fetal Bovine Serum (Gibco), Penicillin/Streptomycin, Amphotericin B (Life Technologies, Eugene, OR, USA), and 2 mM glutamine (complete medium). After NHDF reached confluency, each well was replaced with fresh medium containing one compound at a concentration of 1 µM, and then infected with 1000 newly egressed tachyzoites from RH strain [29]. Screening at 1 μM is recommended by MMV. As a control, infected cells were incubated with complete medium and 0.01% DMSO [29]. The plates were then kept in an incubator at 37 °C in a humid atmosphere with 5% CO_2_ for seven days. After this time, cultures were fixed with 70% ethanol and stained with crystal violet to observe the plates. The area of destruction of the plaques was quantified using the ImageJ^®^ software (version 1.52e) and then the treated cultures were compared with the untreated cultures (control) to determine the percentage of destruction.

Compounds that were able to inhibit plaque formation in more than 80% after treatment with 1 µM were then accessed for IC_50_ determination. For that, 12-well plates containing a monolayer of NHDF were infected with 600 tachyzoites of the RH strain of *T. gondii* and treated with different concentrations for seven days. IC_50_s of compounds were calculated using GraphPad Prism 8.0 software.

### 2.3. Cytotoxicity Assay

The cytotoxic effect of the most active compounds was evaluated through MTS assay as previously described [12,30]. For that, 96-well tissue plates containing NHDF cells were treated with different concentrations of compounds up to 10 µM for seven days. Control cells were incubated with 0.5% DMSO. At the end of the treatment, cell viability was then analyzed through the colorimetric method of MTS (Promega, Madison, WI, USA). The Cytotoxic Concentration of 50% (CC_50_) for the host cells was calculated as for IC_50_ and the Selective Index (SI) was calculated as the ratio of CC_50_/IC_50_.

### 2.4. Forty-Eight-Hour Antiproliferative Assay

NDHF cell monolayers were plated in a 24-well tissue plate containing coverslips, and after confluency was reached, cells were infected with a ratio of 5:1 parasite/host cells with freshly egressed RH strain tachyzoites for two hours. Then, cells were washed twice with phosphate-buffered saline, pH 7.4 (PBS), to remove non-invaded parasites and incubated with a complete medium for two hours more. After this time, infected cells were treated for 48 h with different concentrations of selected compounds, based on the IC_50_ obtained through plaque assay. At the end of the experiment, the cells were washed with PBS, stained with Bouin for 10 min, washed with 70% alcohol, and stained with solutions 2 and 3 of the Fast Panoptic kit for 60 and 10 s, respectively. Two hundred infected cells were quantified per slide using the 100× objective of a Leica DM500 optical microscope, stratifying the number of infected and non-infected cells and the amount of tachyzoite in each infected cell. The proliferation index is the ratio of the product of the total amount of tachyzoites and the percentage of infected cells by the total number of cells [31].

### 2.5. Post-Treatment Recovery (Washout) Assay

NHDF monolayers in 12-well plates were infected with 5000 newly egressed tachyzoites from RH strain and then treated for 3 days with drug concentrations of 0.001 to 1 µM. After treatment, the medium with drugs was removed, and the cells were washed twice and incubated with a fresh medium without drugs for an additional 7 days to allow the remaining parasites (if still viable after treatment) to grow. The plates were then kept in an incubator at 37 °C in a humid atmosphere with 5% CO_2_ throughout the treatment.

### 2.6. Ultrastructural Analysis through Transmission Electron Microscopy (TEM)

T-25 cm^2^ flasks for cell culture with confluent monolayers of NHDF were infected with freshly egressed tachyzoite in a ratio of 10:1 parasite per cell for 2 h of interaction. The flasks were washed and fresh RPMI was added for another two hours. After this, the compounds were added and infected cells were treated for 48 h. The sample fixation and processing for microscopy were performed as previously described [12]. The thin sections of the material were observed in a Fei TecNai G2 120 kV Spirit Electron Microscope at UFMG Microscopy Center.

### 2.7. Immunofluorescence Assay

NHDF cells infected with tachyzoites of *T. gondii* at a ratio of 5:1 of parasites to host cells were treated with 1 µM clindamycin, MMV 1634391, and retapamulin for 24 h. After treatment, cells were fixed with 3.7% freshly prepared formaldehyde, and prepared as previously described [32]. Anti-CPN60 (kindly provided by Dr. Boris Striepen, University of Georgia, USA) was used to label the apicoplast luminal protein CPN60 at a dilution of 1:2000. DAPI (5 µg/mL; Sigma-Aldrich) was used to label the DNA. After labeling, the coverslips were mounted onto slides using Prolong gold (Life Technologies, Eugene, OR, USA), and samples were examined on an Invitrogen EVOS fluorescence microscope.

### 2.8. Statistical Analysis

Data were analyzed using GraphPad Prim 8.0 software. IC_50_ and CC_50_ calculations were performed by fitting the values of proliferation/viability in percentage to a non-linear curve followed by dose–response inhibition analysis through log(inhibitor) vs. normalized response.

## 3. Results

### 3.1. The Pandemic Box Compounds That Showed High Activity and Selectivity against T. gondii Tachyzoites

For the preliminary screening of the 400 compounds, NHDF infected with tachyzoites of *Toxoplasma gondii* were treated for seven days with 1 µM. Twenty-three compounds inhibited *T. gondii* proliferation in more than 50% (Supplemental Figure S1) and were selected for IC_50_ and cytotoxicity studies. The compounds Trimetrexate (MMV1580173), MMV1580844, and clindamycin (MMV000051) were the most active, inhibiting *T. gondii* proliferation with IC_50_s with values lower than 10 nM (Table 1). MMV1593541, MMV1634391, Erythromycin (MMV003137), Retapamulin (MMV1633674), verdinexor (MMV1580493), MMV1782115, URMC-099-C (MMV1580482), and MMV019724 were also highly active against *T. gondii*, presenting IC_50_s under or at the 100–200 nM range (Table 1). The cytotoxicity assay showed that most compounds are highly selective against *T. gondii*, and the SI ranged from 4 to 7447 (Table 1). The compounds with an IC_50_ lower than 5 nM (Trimetrexate, MMV1580844, Clindamycin) were those with the highest SI. Overall, all 23 compounds were shown to be selective, and only four showed a previous activity reported for *T. gondii* (Table 1).

### 3.2. Forty-Eight-Hour Inhibition Assay

**Figure 1:**
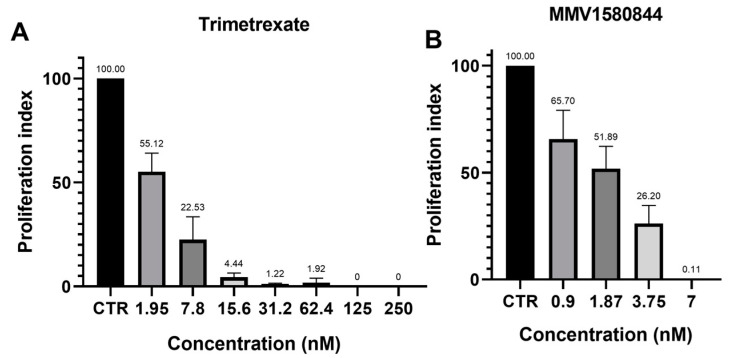
(**A**,**B**) Antiproliferative assay in NHDF cells infected with 5:1 tachyzoites of the RH strain of *T. gondii* after 48 h of treatment. (**A**) Proliferation index of Trimetrexate (MMV1580173) at different concentrations (nM). (**B**) Proliferation index of MMV1580844 at different concentrations (nM). Values represent mean ± SD of three experiments. CTR = Control.

**Figure 2:**
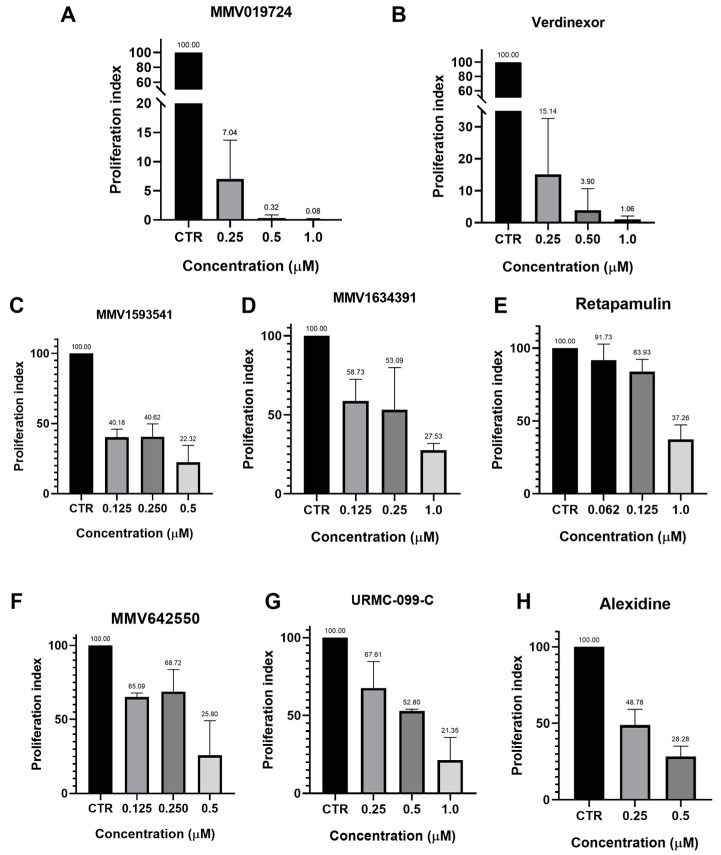
(**A**–**H**) Antiproliferative assay in NHDF cells infected with 5:1 tachyzoites of the RH strain of *T. gondii* after 48 h of treatment on a µM. (**A**) Proliferation index of MMV019724. (**B**) Proliferation index of verdinexor (MMV1580493). (**C**) Proliferation index of MMV1593541. (**D**) Proliferation index of MMV1634391. (**E**) Proliferation index of Retapamulin (MMV1533674). (**F**) Proliferation index of MMV642550. (**G**) Proliferation index of URMC-099-C (MMV1580482). (**H**) Proliferation index of Alexidine (MMV396785). Values represent the mean ± SD of three experiments. CTR = Control.

Considering the compounds with the best inhibition activity against *T. gondii*, we carried out a study to verify which ones could inhibit *T. gondii* proliferation in the first lytic cycle of development (48 h of infection for the RH strain of *T. gondii*). Trimetrexate (MMV1580173) and MMV1580844 inhibited *T. gondii* proliferation by approximately 50% at concentrations close to 2 nM. Treatment of infected cells with 7 nM MMV1580844 and 15 nM trimetrexate inhibited the tachyzoite proliferation in more than 90% (Figure 1A,B). Compounds MMV019724 (Figure 2A) and verdinexor (MMV1580493) (Figure 2B) inhibited proliferation by more than 80% in a concentration of 0.25 µM, and 1 μM inhibited parasite proliferation in 99%. Alexidine and MMV1593541 inhibited around 60% of parasite proliferation in a concentration of 0.250 µM (Figure 2C,H), and 0.5 µM MMV642550 (Figure 2F) inhibited around 75% of *T. gondii* proliferation after 48 h of treatment. MMV1634391 (Figure 2D), Retapamulin (Figure 2E), URMC-099-C (Figure 2G), Eberconazole, Triapine, MMV1782115, RWJ67657, and DNDI147411 (Supplemental Figure S2) inhibited proliferation by more than 60% with 1 μM.

### 3.3. Ultrastructural Analysis after 48 h of Treatment

To better understand the mode of action of the most active compounds, we evaluated the ultrastructural alterations on tachyzoites of *T. gondii* caused after treatments with 0.25 µM verdinexor, 0.25 µM MMV019724, 0.5 µM MMV1593541, 1 µM Eberconazole, 1 µM MMV642550, 1 µM RWJ-67657, 1 µM URMC-099-C, and 3 nM MMV1580844 through transmission electron microscopy.

**Figure 3:**
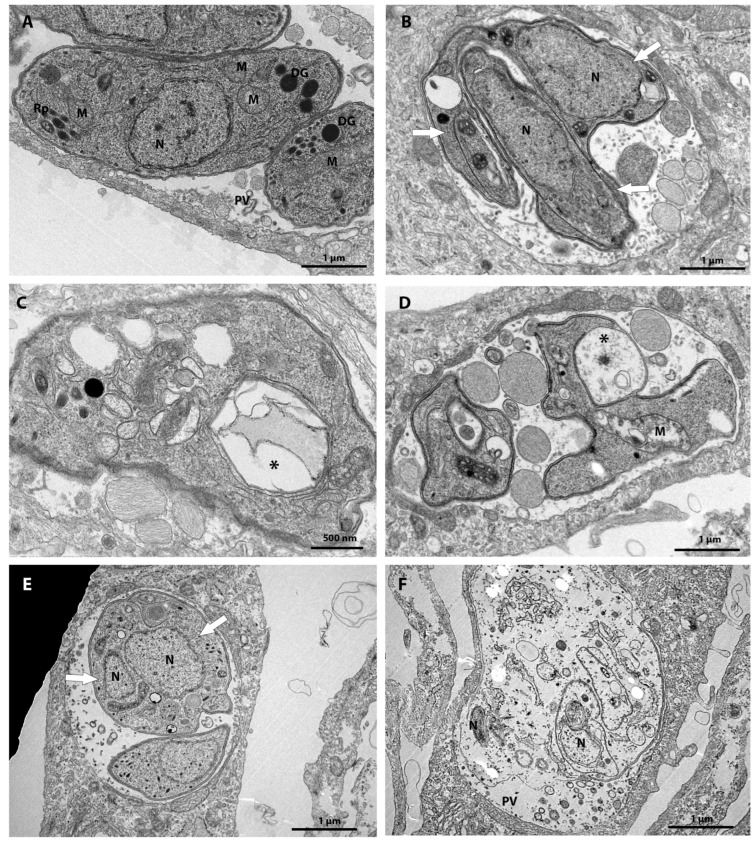
(**A**–**F**) Transmission electron microscopy analysis of *T. gondii* tachyzoites treated for 48 h with different compounds from the Pandemic Box. (**A**) Untreated tachyzoites show normal morphology. (**B**,**C**) Parasites treated with 0.25 μM verdinexor (MMV1580493) affected the parasite division process, (**B**) evidenced by the presence of three daughter cells (white arrows) possibly resulting from the interruption of the parasite cell division process, (**C**,**D**) and induced extensive vacuolization (asterisk) in the parasite. (**D**) Parasite treated with 0.25 μM verdinexor shows mitochondrial (M) swelling and pellicle alteration. (**E**) Parasites treated with the compound MMV019724 at a concentration of 0.25 μM showed defects in the cell division process; it is possible to see two nucleus profiles in a single cell (top of the Parasitophorous Vacuole) (arrow white). (**F**) Treatment with 0.25 µM induced parasite lysis. DG—Dense granules; M—Mitochondria; N—Nucleus; Rp—Rhoptries; PV—Parasitophorous Vacuole.

Figure 3A presents the ultrastructure of an untreated parasite with typical morphology. Treatment of tachyzoites *T. gondii* with 0.25 µM verdinexor for 48 h caused a drastic effect on the division of the parasite (Figure 3B). It is possible to observe three daughter cells (large arrows) resulting from the interruption of the parasite’s cell division process. In addition, treatment with 0.25 µM verdinexor also caused extensive vacuolization (asterisks in Figure 3C,D) and induced mitochondrial alterations (M in Figure 3D). Treatment with 0.25 µM MMV019724 affected the parasite cell division process, evidenced by the presence of profiles of two nuclei in the same parasite with a rounded shape (Figure 3E), and induced a lytic effect in the parasite (Figure 3F).

**Figure 4:**
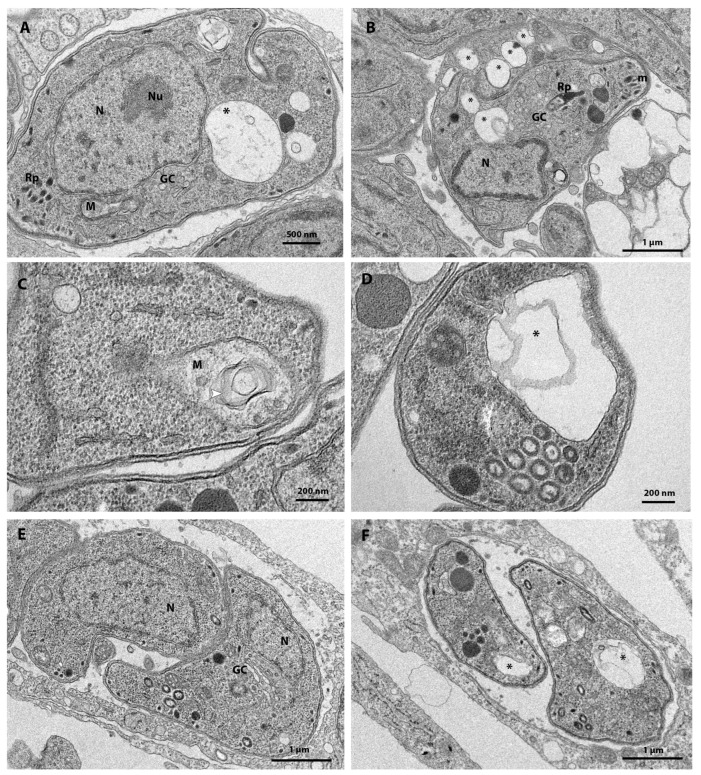
(**A**–**F**). Transmission electron microscopy analysis of *T. gondii* tachyzoites treated for 48 h with different compounds from the Pandemic Box. (**A**,**B**) Parasites treated with 0.5 µM MMV1593541 showed defects in parasite cell division and the formation of vacuoles in the parasite (asterisks). (**C**) Parasites treated with 1 µM Eberconazole showed mitochondrial swelling (white arrow head) and (**D**) extensive vacuoles in the parasite (asterisk). (**E**) Parasites treated with 1 µM MMV642550 showed changes in the Golgi complex and (**F**) extensive vacuolization (asterisks). GC—Golgi complex; M—Mitochondrion; m—Micronemes; N—Nucleus; Nu—Nucleolus; Rp—Rhoptries.

**Figure 5:**
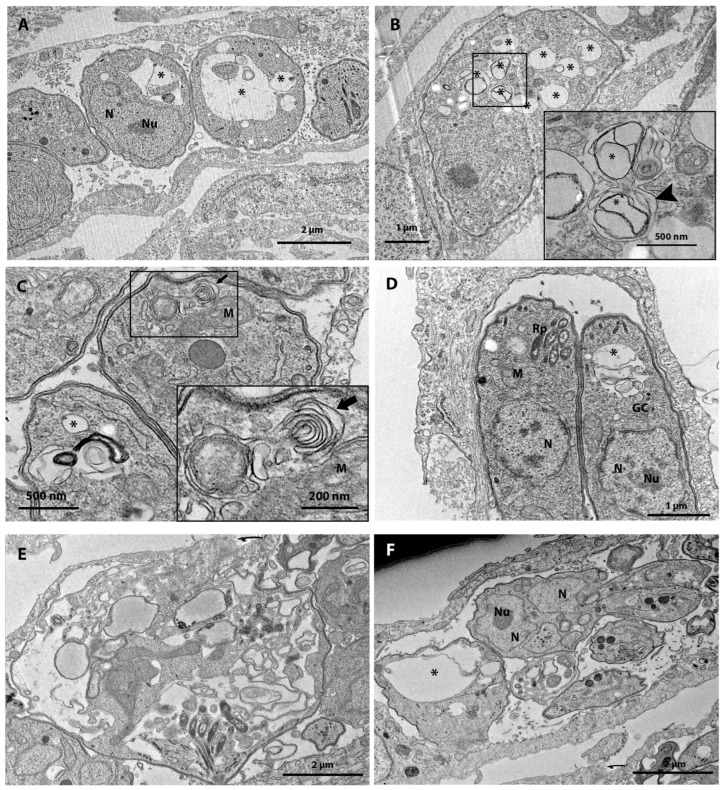
(**A**–**F**). Transmission electron microscopy analysis of *T. gondii* tachyzoites treated for 48 h with different compounds from the Pandemic Box. (**A**,**B**) Parasites treated with 1 µM RWJ-67657 (MMV1580797) showed intense vacuolization (asterisks). In the inset (**B**), it is possible to observe vacuoles in detail (black arrowhead). (**C**,**D**) Parasites treated with 1 µM URMC-099-C showed mitochondrial alteration, vacuolization (asterisks), and myelin-like body (inset—black arrow). (**E**,**F**) Parasites treated with 3 nM MMV1580844 showed complete ultrastructural alteration of the parasite, vacuoles (asterisk), and (**F**) defect in the cell division process. GC—Golgi complex; M—Mitochondria; N—Nucleus; Nu—Nucleolus; Rp—Rhoptries.

Treatment with 0.5 µM MMV1593541 induced the formation of extensive cytoplasm vacuolization (asterisks, Figure 4A), and affected cell division (Figure 4B). Treatment with 1 µM Eberconazole induced mitochondrial swelling (white arrowhead) and extensive vacuolization (Figure 4C,D), and 1 µM MMV642550 affected the Golgi complex (GC) (Figure 4E) and induced extensive vacuolization inside the parasite body (asterisk in Figure 4F). Treatment with 1 µM RWJ-67657 or URMC-099-C also induced an extensive vacuolization (asterisks) in *T. gondii* and myelin-like bodies (arrowhead) at the parasite cytoplasm (Figure 5A–D). The inset of Figure 5C also shows mitochondrial alteration after treatment with URMC-099-C. MMV1580844 was the most active compound and treatment with 3 nM of this drug caused parasite lysis (Figure 5E). MMV1580844 treatment also disrupted the parasite division process, as evidenced by a large cell presenting interrupted daughter cell budding and several nucleus profiles (Figure 5F).

### 3.4. MMV163439 and Retapamulin Treatment Induce Apicoplast Loss in T. gondii

**Figure 6:**
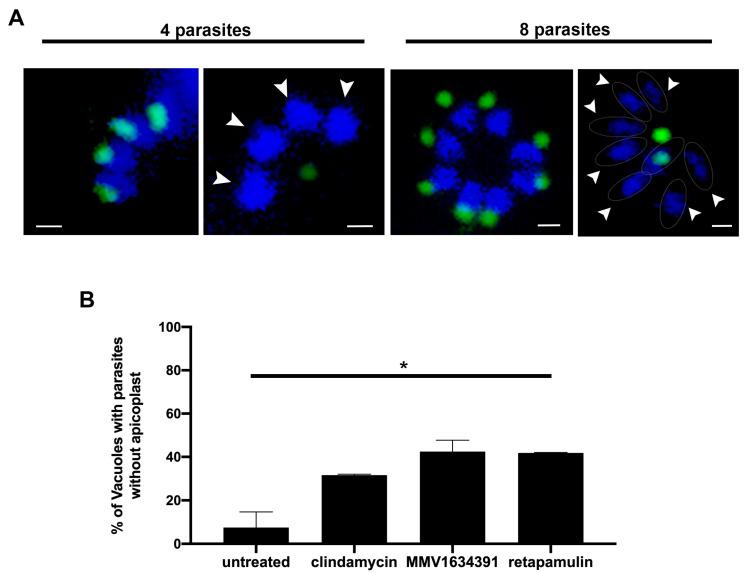
NHDF cells infected with tachyzoites of *T. gondii* were treated for 24 h with 1 µM clindamycin, MMV1634391, and retapamulin. Parasites were labeled with anti-CPN60 for the apicoplast (green) and DAPI for DNA (blue). Vacuoles containing 4 or 8 parasites (**A**) were analyzed and those showing at least one parasite without apicoplast were scored (white arrowheads point parasites without apicoplast) (**B**). Results are the mean ± standard deviation of two independent experiments. Scale bar = 1 µm. * *p* < 0.05 untreated compared to treated parasites.

Antibacterial inhibitors of prokaryote protein synthesis and DNA replication or maintenance, such as clindamycin and fluoroquinolones, respectively, are known to cause apicoplast loss in *T. gondii* [37,38]. Within the new compounds identified in this study, MMV163439 and retapamulin are antibacterials designed to bind to the DNA minor groove and inhibit prokaryote protein synthesis, respectively [39,40]. Thus, we evaluated the effect of both compounds in the maintenance of apicoplast by tachyzoites after 24 h of treatment. For that, we scored vacuoles of 4 or 8 parasites (Figure 6A) containing at least one parasite presenting apicoplast loss (arrowheads in Figure 6A). While the vacuoles from untreated parasites showed a frequency of apicoplast loss lower than 10%, vacuoles of parasites treated with clindamycin, MMV163439, and retapamulin showed a frequency of loss higher than 30% (*p* < 0.05 compared to untreated).

### 3.5. MMV1580844 and Trimetrexate Post-Treatment Recovery Assay

**Figure 7:**
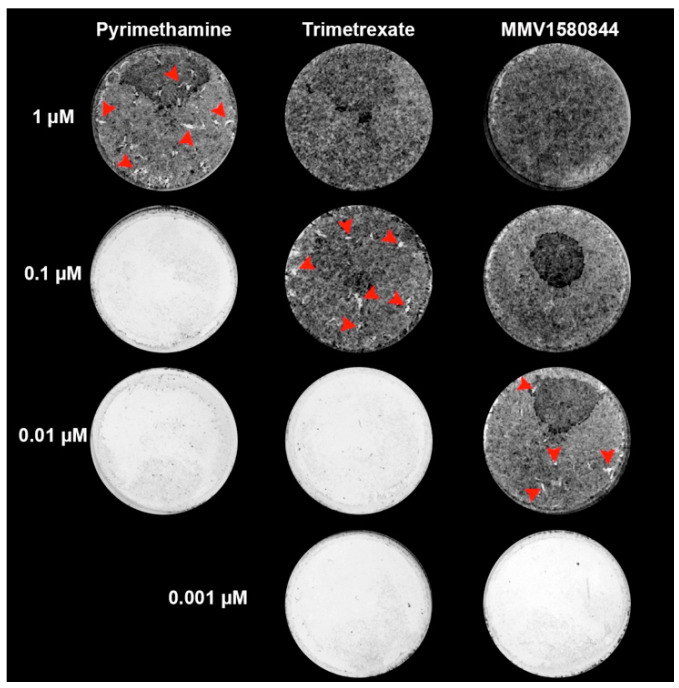
Post-treatment recovery assay of *T. gondii* tachyzoites from RH strain after treatment with pyrimethamine (PYR), trimetrexate, and MMV1580844. Parasites were treated for 3 days with the drugs and then allowed to recover for 7 days in fresh medium without drugs. Monolayer lysis and plaque areas (arrowheads) were seen for all concentrations of PYR, 0.001–0.1 µM trimetrexate, and 0.001–0.01 µM MMV1580844. No plaques were seen for 1 µM trimetrexate and 0.1–1 µM MMV1580844, indicating that treatment with these compounds possibly led to a complete eradication of infection.

Within the most active compounds are two inhibitors of dihydrofolate reductase (DHFR): MMV1580844 and trimetrexate. While the effect against *T. gondii* was already reported for trimetrexate, this is the first report for MMV1580844. Considering the potent anti-proliferative effect of this new drug and that DHFR is a validated target for the treatment of toxoplasmosis, we compared the effect of this new drug with trimetrexate and pyrimethamine in a washout assay. Treatment with PYR could not eradicate *T. gondii* proliferation, as plaques were still visible even when infected cells were treated with 1 µM. Treatment with trimetrexate abolished parasite proliferation recovery at 1.0 µM, but plaques were visible at 0.1 µM treatment. MMV1580844 abolished parasite proliferation recovery at 0.1 µM, and plaques were observed only when infected cells were treated with 0.001 and 0.01 µM (Figure 7).

## 4. Discussion

To analyze the possible anti-*Toxoplasma gondii* activity of the Pandemic Box compounds, the screening was carried out using a concentration of 1 μM, as recommended by the MMV. A total of 23 compounds were selected, which inhibited the proliferation of *T. gondii* by more than 50% (Figure S1). Of these, three belong to the antifungal class, eleven are antibacterials, and nine are antivirals. It is also worth noting that this is the first report of the anti-*T. gondii* activity of 19 of the selected compounds (Table 1). Four compounds have exhibited previous anti-*T. gondii* activity. The discovery of these compounds brings new perspectives for the treatment of toxoplasmosis, since the chemotherapy used is related to adverse events and parasite resistance [6,10,11]. During the submission process of this work, another paper showing the anti-*T. gondii* activity of the Pandemic Box compounds was accepted for publication [41]. According to this, 42 compounds showed a Half Effective Concentration (EC_50_) ranging from 2.4 to 913.1 nM. From these 42 compounds, we confirmed the activity of 20 in this paper; indeed, we also identified the compounds tafenoquine, MMV1578890, and Fenretinide as active against the tachyzoites of *T. gondii.*

Of the 23 compounds identified in this present work, the most active were antibacterial inhibitors of validated targets against *T. gondii*: clindamycin, trimetrexate, and MMV1580844. While the first targets the apicoplast protein synthesis and is already used in clinics for toxoplasmosis treatment, the last two are inhibitors of DHFR [27,37,38,39]. Trimetrexate is an anticancer agent available on the market, and its effect against *T. gondii* was previously reported [34,42], but due to its higher selectivity for human DHFR when compared to the parasite’s enzyme, its use as an anti-parasitic is not recommended, due to toxicity. However, this is the first report of the effect of the non-commercial antibacterial drug MMV1580844 against *T. gondii*. In vitro studies showed that this drug acts against the parasite with concentrations close to 1 nM (Table 1 and Figure 1B) and has an SI of 7447. Previous screening with Pandemic Box compounds also showed that MMV1580844 was highly active against amoebae species [43]. TEM analysis of tachyzoites treated for 48 h with only 3 nM MMV1580844 showed that this drug affects the parasite division process and also induces parasite cell death. Indeed, the washout assay showed that MMV1580844 could irreversibly inhibit tachyzoite proliferation with 0.010 µM, and similar results were observed with 0.1 µM trimetrexate, but concentrations of PYR up to 1 µM could not ablate the parasite proliferation (Figure 6). These results show that MMV1580844 is 10 times and 100 times more active than trimetrexate and PYR, respectively, against *T. gondii* and is potentially a candidate drug for toxoplasmosis treatment.

Our screening also identified three other new antibacterial drugs highly active against *T. gondii*: retapamulin, MMV1634391, and MMV1593541. All of them inhibited tachyzoite proliferation with an IC_50_ lower than 100 nM and showed selectivity for parasites over host cells (Table 1). MMV1634391 was designed to bind to and showed to bind to *Trypanosoma brucei* nuclear and kinetoplast DNA [39,44]. In addition, in silico studies showed a potential antimalarial activity through its binding to the Sodium ATPase (PfATP4), which is a cytosolic enzyme, exclusive to parasites of the phylum Apicomplexa, which is responsible for maintaining Na^+^/H^+^ regulation, helping to maintain the acid load of the parasite [45]. Thus, our study is the first report of in vitro activity of MMV1634391 with a parasite of the apicomplexa phylum. Retapamulin (MMV1633674) is an antibacterial inhibitor of protein synthesis that also has shown antiviral activity against *Ebola* [46]. MMV1634391 and retapamulin induced apicoplast loss in tachyzoites, suggesting that they could bind to this organelle DNA and inhibit its protein synthesis, respectively (Figure 6). Drugs that affect the apicoplast protein synthesis and DNA maintenance are known to exert a delayed effect on the parasite, causing the inhibition of proliferation only at the second lytic cycle of the parasite [47]. MMV1634391 and retapamulin inhibited tachyzoite proliferation in more than 50% after treatment with 1 µM for 48 h (Figure 2D,E); thus, we cannot exclude that they could affect other targets in the parasite.

MMV1593541 is a quinolinyl pyrimidine derivative known to be an inhibitor of type II NADH dehydrogenase (NDH-2) [48], an enzyme responsible for the entry of NADH into the electron transport chain in the mitochondrion and that is essential for the maintenance physiology in *T. gondii* [49,50]. The forty-eight-hour inhibition assay showed that this compound is also highly effective against *T. gondii*, inhibiting its proliferation by more than 50% with a concentration of 125 nM. MET analysis confirmed the direct effect of MMV1593541 on *T. gondii,* causing extensive vacuolization in the parasite cytoplasm (asterisk) (Figure 4A,B).

Our screening also identified compounds of the antiviral class able to inhibit *T. gondii* proliferation in more than 50% with 1 µM and with low toxicity for NHDF cells with tested concentrations. Verdinexor, MMV019724, URMC-099-C, and MMV1782115 were the most active and affected parasite proliferation with IC_50_s at the 100 nM (sub)order. Verdinexor is active against *Epstein–Barr* virus (EBR) and *Human cytomegalovirus* (HCMV) [51,52], and the compound MMV019724 has already been described as active against the pathogens *Madurella* spp., *Sporothrix* spp., *Cryptococcus* spp., and *Candida auris* [53,54,55]. URMC-099C was reported active against the amoeba *Balamuthia Mandrillaris* with an IC_50_ of 5.35 μM [43]. Ultrastructural analysis through TEM showed that URMC-099-C, verdinexor, and MMV019724 exert a direct effect on *T. gondii*, inhibiting its division process and inducing cell death processes. This is the first report of the anti-*T. gondii* activity of all four compounds.

MMV642550 and RWJ-67657 are two other antivirals that also showed a promising effect against *T. gondii*. RWJ-67657 is a pyridinylimidazole inhibitor of p38 MAPK [56], and its anti-*T. gondii* activity was previously described [33]. This work reported an IC_50_ of 0.8 and 5 μM for Me49 and RH strains, respectively; our work shows an inhibition of 59.5% with 1 µM and IC_50_ of 411.7 nM after 48 h and 7 days of treatment of the RH strain, respectively, which is similar to the results previously obtained for Me49. Recent work also showed the potential effect of RWJ-67657 against tissue cysts [41]. MMV642550 is a benzimidazole that has been recently reported as active against *Plasmodium falciparum* at a submicromolar concentration. In silico analysis showed that the glideosome component GAP50 is a possible drug target of MMV642550 [57]. TEM analysis showed that as the other antivirals analyzed in this work, MMV642550 and RWJ-67657 also induced an extensive vacuolization in *T. gondii*.

Our analysis also identified three antifungal agents able to inhibit *T. gondii* proliferation with submicromolar concentrations (Table 1, Figure 2H and Figure S2A). The anti-*T. gondii* activity of compounds primarily developed to treat fungal infections has been reported previously [58,59,60]. However, this is the first report for alexidine, ciclopirox, and Eberconazole (Table 1). Alexidine (MMV396785) is a biguanide, a known inhibitor of DHFR [61], the same target of PYR, as well as trimetrexate and MMV1580844, also analyzed in this study. Alexidine also showed anti-amoebicidal activity against *Acanthamoeba castellanii* and *A. polyphaga* [62]. Ciclopirox is a broad-spectrum antifungal agent that also shows anticancer activity [63]. Eberconazole is an antifungal drug of the imidazole class and acts in inhibiting the ergosterol synthesis mediated by cytochrome P-450 [64]. Although *T. gondii* does not synthesize any kind of sterol, inhibitors of its synthesis, such as itraconazole and fluconazole, show activity against this parasite [45,46]. Eberconazole was also active against amoebae species [43], *Madurella mycetomatis* (IC_50_ 0.72 μM) [53], *Sporothrix brasiliensis*, *S. globosa*, and *S. schenckii* [54]. TEM analysis confirmed that Eberconazole directly acts against *T. gondii*, inducing parasite vacuolization and mitochondrial swelling (Figure 4C,D).

In conclusion, our screening identified 19 new, promising drugs/compounds active against the tachyzoite stage of *T. gondii* at the submicromolar concentration, and six of them showed IC_50_ lower than 100 nM and should be explored for in vivo analysis in the future.

## Figures and Tables

**Table 1:** Activity and selectivity of Pandemic Box compounds against *Toxoplasma gondii*.

Compound Identification				Previous Activity Reported against *T. gondii*?	^a^ IC_50_ (nM) in Tachyzoites of RH Strain (7 Days)	Cytotoxicity against NHDF (7 Days)	
Plate Position	MMV ID	Trivial Name	Disease Area			^b^ CC_50_ (nM)	^c^ SI
A-A2	MMV1634492	Eberconazole	Antifungal	No	723.6 ± 12.6	^d^ ND	ND
A-A3	MMV002731	Ciclopirox	Antifungal	No	497.4 ± 18.1	4000	8
A-H3	MMV396785	Alexidine	Antifungal	No	271.9 ± 38.4	1627	6
A-F9	MMV1580173	Trimetrexate	Antibacterial	Yes [33,34]	3.9 ± 0.1	11,880	3046
A-F10	MMV000043	Tafenoquine	Antibacterial	No	896.9 ± 0.1	16,270	18
A-G10	MMV003137	Erythromycin	Antibacterial	Yes [35]	131.4 ± 15.8	11,930	91
B-E5	MMV1578890	MMV1578890	Antibacterial	No	1100.0 ± 0.1	10,300	9
B-B9	MMV1593541	MMV1593541	Antibacterial	No	37.9 ± 40.7	2541	67
B-C9	MMV1593537	MMV1593537	Antibacterial	No	940.0 ± 38.6	5657	6
B-D10	MMV1580844	MMV1580844	Antibacterial	No	1.5 ± 0.1	11,170	7447
C-C2	MMV1634391	MMV1634391	Antibacterial	No	90.6 ± 39.6	ND	ND
C-D2	MMV1633674	Retapamulin	Antibacterial	No	37.3 ± 14.3	9979	270
C-F5	MMV1634399	MMV1634399	Antibacterial	No	920.3 ± 0.1	24,120	26
C-G5	MMV000051	Clindamycin	Antibacterial	Yes [29,32]	3.3 ± 1.9	8841	2679
D-E4	MMV642550	MMV642550	Antiviral	No	320.0 ± 17.0	6730	21
D-B6	MMV001793	Fenretinide	Antiviral	No	918.1 ± 29.1	26,670	29
D-F8	MMV1580797	RWJ-67657	Antiviral	Yes [36]	411.7 ± 21.7	12,620	31
D-A10	MMV1782115	MMV1782115	Antiviral	No	160.6 ± 60.8	9429	58
E-D4	MMV098836	DNDI1417411	Antiviral	No	485.2 ± 43.8	14,510	32
E-H5	MMV1580493	Verdinexor	Antiviral	No	70.0 ± 24.4	2862	63
E-A6	MMV1580482	URMC-099-C	Antiviral	No	45.4 ± 31.9	ND	ND
E-C6	MMV1580496	Triapine	Antiviral	No	717.7 ± 0.1	2795	4
E-H7	MMV019724	MMV019724	Antiviral	No	109.1 ± 47.6	2500	20

^a^ Half Inhibition Concentration (IC_50_) against *T. gondii* tachyzoites of two independent experiments. ^b^ Half Cytotoxic Concentration (CC_50_) against NHDF cells of two independent experiments. ^c^ Selectivity Index, calculated based on the CC_50_ NDHF cells/IC_50_ *T. gondii* ratio. ^d^ ND = not determined, host cells showed 100% of viability after treatment with concentrations up to 10 µM of compounds.

## Data Availability

The original contributions presented in this study are included in the paper. Further inquiries can be directed to the corresponding author.

## Supplementary Materials

The following supporting information can be downloaded at https://www.mdpi.com/article/10.3390/tropicalmed8120510/s1. Figure S1: Preliminary screening of the 400 drugs and drug-like compounds contained in the Pandemic Box; Figure S2: Antiproliferative assay in NHDF cells infected with 5:1 tachyzoites of the RH strain of *T. gondii* after 48 h of treatment in increasing concentrations on a micromolar scale.
