# Supplementary material for: Medicines for Malaria Venture Pandemic Box In Vitro Screening Identifies Compounds Highly Active against the Tachyzoite Stage of Toxoplasma gondii
Source: Trop Med Infect Dis. 2023 Nov 29;8(12):510. doi: 10.3390/tropicalmed8120510 (PMC10747034; doi:10.3390/tropicalmed8120510)
Supplement: Supplementary file 1 [file tropicalmed-08-00510-s001.zip › tropicalmed-2705888-supplementary.pdf]

Supplementary Figure S1

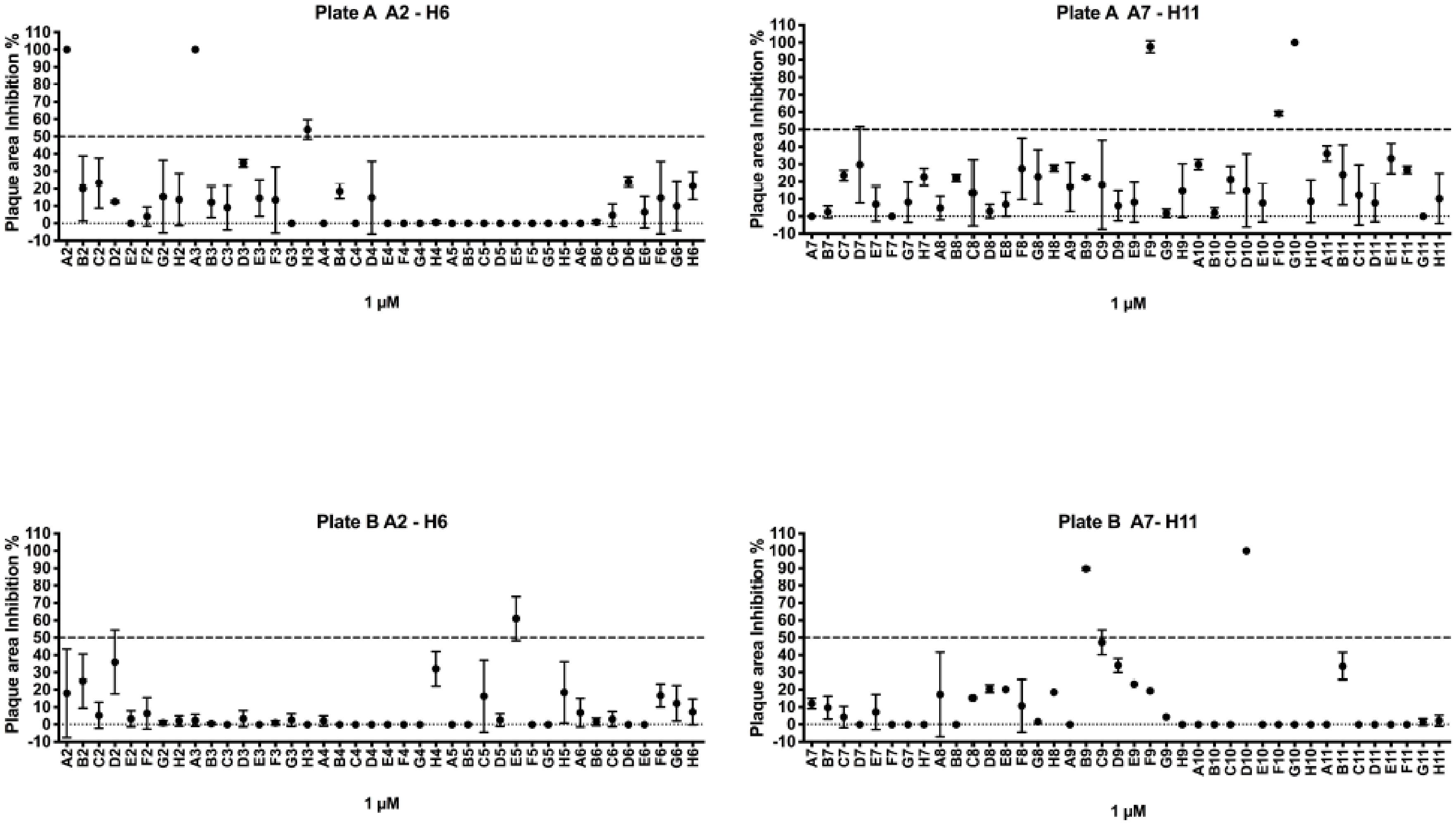

Supplementary Figure S1 continuation

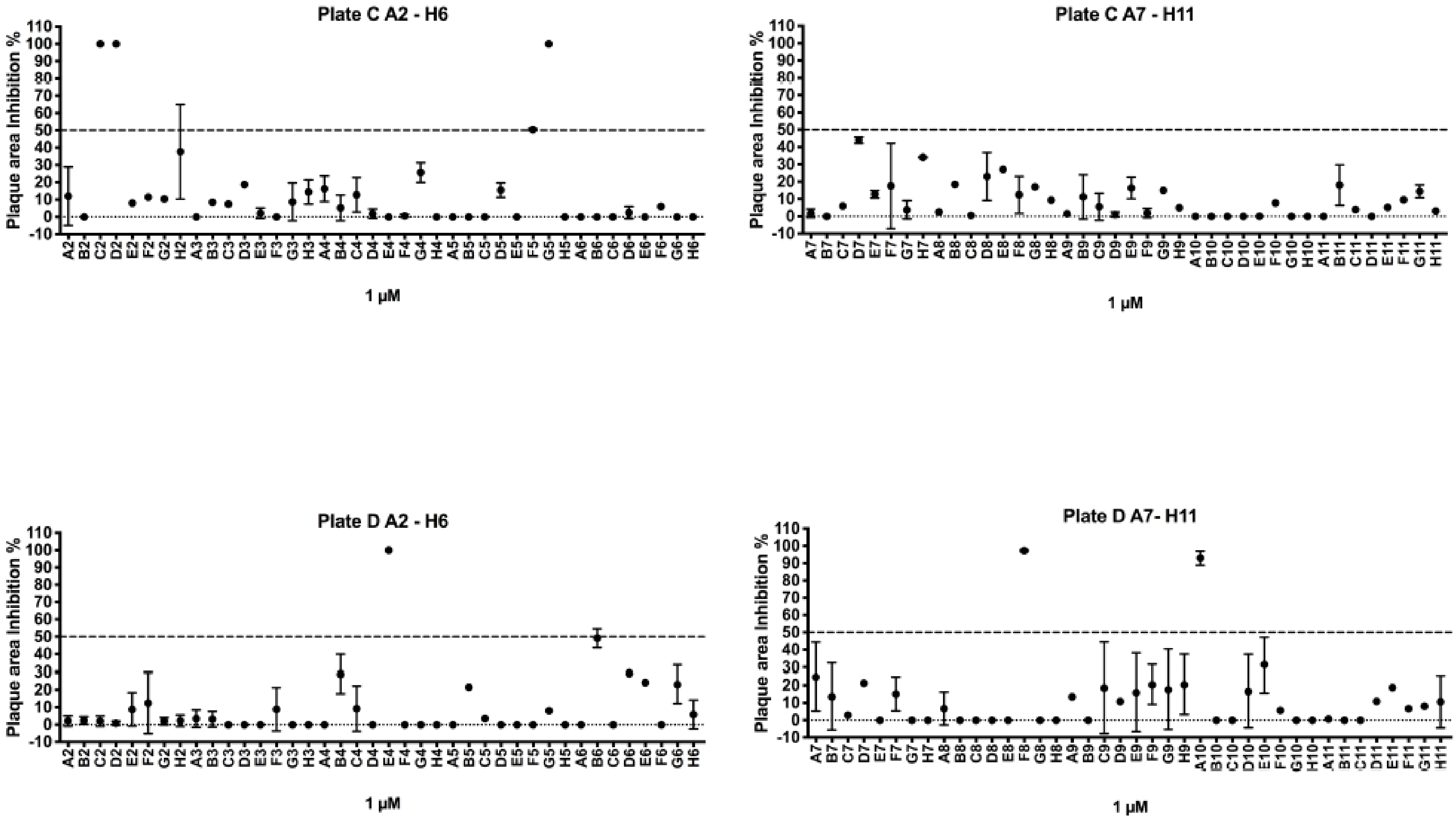

Supplementary Figure S1 continuation

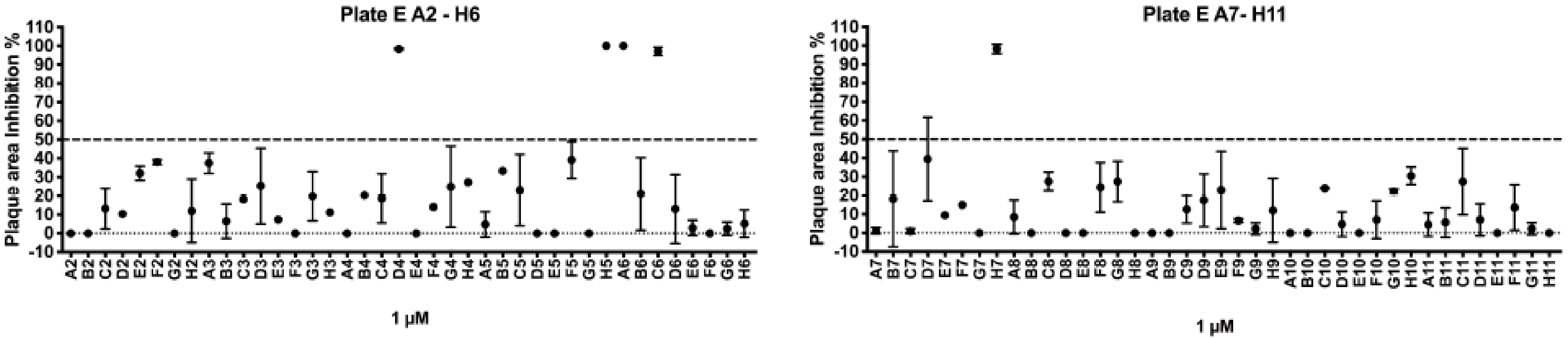

Figure S1. Preliminary screening of the 400 drugs and drug-like compounds contained in the Pandemic box. NDHF monolayers in 6-well plates were infected with 1,000 tachyzoites of T. gondii and treated for 7 days with compounds. The inhibitory activity was evaluated by measuring the percentage of monolayer destruction compared to the untreated group. Results represent the mean ± standard deviation of two independent experiments.

Supplementary Figure S2

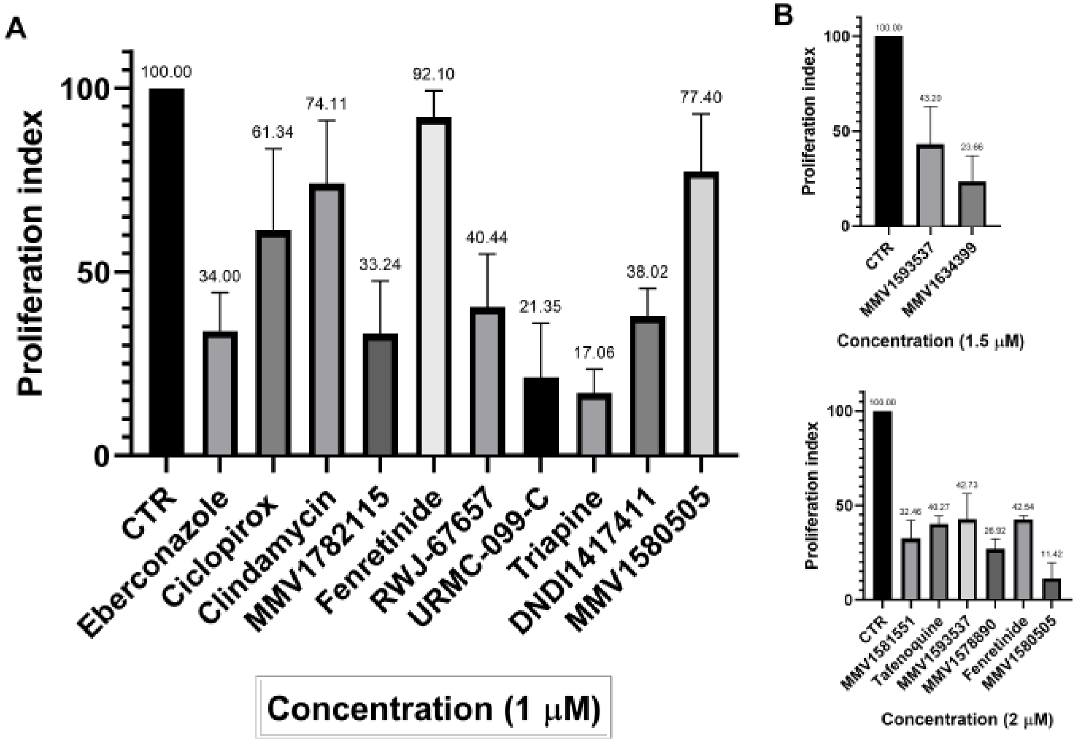

Figure S2. A-C. Antiproliferative assay in NHDF cells infected with 5:1 tachyzoites of the RH strain of T. gondii after 48 hours of treatment in increasing concentrations on a micromolar scale. A. Proliferation index of selected compounds from Pandemic Box at 1 μM concentration. B. Proliferation index of selected compounds from Pandemic Box at 1.5 μM concentration. C. Proliferation index of selected compounds from Pandemic Box at 2 μM concentration. Values represent mean ± SD of three experiments. CTR= Control.
